# Effect of COVID-19 pandemic on blood transfusion service: an experience from a regional blood transfusion center

**DOI:** 10.1097/BS9.0000000000000161

**Published:** 2023-05-24

**Authors:** Sanjay K. Thakur, Anil K. Sinha, Dinesh K. Negi, Sompal Singh

**Affiliations:** a PhD Research Scholar, P. G. Department of Zoology, Veer Kunwar Singh University, Ara, Bihar 802301, India; b Dean Faculty of Science and Head P. G. Department of Zoology, Veer Kunwar Singh University, Ara, Bihar 802301, India; c Former CMO In charge, Department of RBTC, Hindu Rao Hospital, Delhi 110007, India; d Specialist Pathologist, Head Department of Regional Blood Transfusion Centre and Department of Pathology, Hindu Rao Hospital, Delhi 110007, India

**Keywords:** Blood bank inventory, Blood components, Blood recipients, Blood transfusion, COVID-19 pandemic

## Abstract

The unforeseen and uncertain life-threatening situation of the COVID-19 pandemic dramatically affected all areas of the human daily work schedule. This study was designed to assess the impact of the COVID-19 pandemic on blood transfusion services and discuss the adopted confrontation measures for uninterrupted blood supply during the pandemic situation. The data on blood donation, blood component preparation, and issue from January 2019 to December 2022 were collected from the inventory registers of the RBTC, Delhi, India. Compared to the non-pandemic year 2019, during the year 2020, all variables decreased gradually. The observed maximum decrease in variables such as blood collection (–79.16%) in the month of October, blood issue (–71.61%) in the month of August, random donor platelets (RDP) preparation (–98.09%) in the month of October, RDP issue (–86.08%) in the month of September, fresh frozen plasma (FFP) preparation (–100%) in the month of October, and FFP issue (–96.08%) in the month of July with an annual decrease of –45.52%, –42.87%, –33.00%, –59.79%, –40.98%, and –54.48%, respectively, as compared to year 2019. Compared to year 2020, in year 2021, the annual increase in blood collection, blood issue, FFP preparation, FFP issue, RDP preparation, and RDP issue was +50.20%, +21.68%, +65.31%, +78.52%, +116.23%, and +213.30%, respectively. Our study results show that the COVID-19 pandemic has significantly affected blood transfusion services at our blood bank. The adopted coping strategies to maintain the safe and uninterrupted blood transfusion chain at our blood bank gave us lessons for future preparedness if faced with a similar situation.

## 1. INTRODUCTION

On January 30, 2020, the novel coronavirus (COVID-19) outbreak was reported as a public health emergency of international concern, and on March 11, 2020, it was declared a global pandemic by the World Health Organization (WHO).^1^ As a precautionary measure, a nationwide lockdown was imposed by the Central and State governments of India to contain the spread of the COVID-19 outbreak.^2^ Although attempts were made to predict the magnitude of the problem,^3^ the situation remains uncertain. This unforeseen and uncertain life-threatening situation dramatically affected all areas of the human daily work schedule, livelihood, economy, and healthcare services worldwide.^4^ There was geographic heterogeneity in the disease burden.^5,6^ COVID-19 pandemic also posed challenges to blood transfusion services in the management of adequate blood inventory to satisfy the blood component demand, recruit non–COVID-19-risk healthy blood donors, and ensure the safety of blood bank staff and blood donors from COVID-19 risk.^7,8^ In India, the general community lacks the motivation and willingness to donate blood,^9,10^ and in the pandemic crisis in particular, there is a great deal of confusion over the safety from the risk of infection during blood donation and transfusion.^11,12^ In addition, to contain the spread of disease, the restriction imposed on people’s movements during the pandemic became a hindrance factor for organizing voluntary blood donation camps and regular voluntary blood donation, even if they wished to donate. As the pandemic evolved in India, blood transfusion centers continued their inventory management after making the necessary changes to their policy of donor recruitment and selection, as per the guidance of the National Blood Transfusion Council (NBTC, India).^8,13^ This study was designed to assess the impact of COVID-19 pandemic on blood donation and blood supply during the pandemic and to compare it with non-pandemic situation of blood transfusion services and discuss the adopted confrontation measures to uninterrupted blood supply during the pandemic situation.

## 2. MATERIALS AND METHODS

### 2.1. Study design and data collection

A cross-sectional study was undertaken from January 2019 to December 2022 at the Regional Blood Transfusion Centre, Delhi, India. The data for 2019 were used as a baseline against which the next 3-year data were compared. Data of blood donors, blood components prepared and blood components issued to recipients, such as the number of packed red blood cells (PRBC), fresh frozen plasma (FFP) and random donor platelets (RDP) or platelet concentrate was included in the present study. Blood donors who participated in the study met all criteria and requirements for blood donation as per Standard Operating Procedure (SOP).

During the pandemic, our hospital was designated as a dedicated COVID care center and the additional COVID-19 cope-up measures were adopted as precautionary measures. As per the slogan of the WHO, “Safe Blood Saves Lives,” on World Blood Donors Day 2020^10^ additional guidelines and recommendations of WHO^11^ and other blood transfusion services governing and accrediting agencies, such as the National Aids Control Organization (NACO, India), the National Blood Transfusion Council (NBTC, India),^9^ American Association of Blood Banks (AABB),^12^ and Centers for Disease Control and Prevention (CDC)^13^ were adopted as precautionary measures to maintain the safe blood transfusion chain.

### 2.2. Data collection and statistical analysis

The study data of blood donation, blood component preparation, and issue (recipients) were collected from the registers of blood bank inventory. Data were entered into the Microsoft Excel sheet and for the descriptive and inferential statistics, an open source Windows statistical software package, Python version 3.0 (Python, Inc., Chicago, Illinois) and Jupyter Notebook version 6.0.3 (Python, Inc.) was used. Study results were presented as total, mean ± standard deviation, minimum, maximum, and percentage in the form of tables and figures. The Mann–Whitney *U* test was used to calculate the significance of the difference in the blood donation, component preparation, and components issued (recipient) between the non-pandemic year 2019 and years 2020, 2021, and 2022. A *P* value of <.05 was considered statistically significant.

## 3. RESULTS

**Figure 1.**
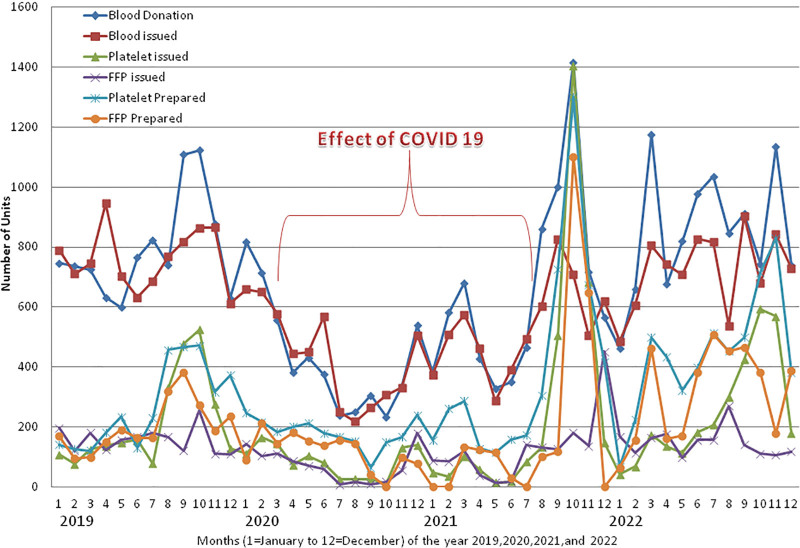
Monthly data of years 2019, 2020, 2021, and 2022 showing differences and effects of COVID-19 in the monthly trend of the number of units of blood donation, blood issued (whole blood and pack cell volume), platelets unit prepared, platelets unit issued, FFP prepared and issued. FFP = fresh frozen plasma.

A comparison of blood bank inventory data for donors, number of requests for blood components, number of cross-matches done, blood component preparation and issue to recipient, across the years 2019 to 2022 was done. Our results showed variations in the characteristics of blood donation and supply between the non-pandemic year 2019 (base line) and the pandemic years 2020, 2021, and 2022 (Table 1 and Fig. 1). Blood bank services were adversely affected after the start and during the COVID-19 pandemic lockdown.

**Table 1:** Data of blood donors, blood recipients, platelet prepared, platelet recipient, FFP prepared, FFP recipient were presented as an annual total (monthly mean ± SD) for the years 2019 to 2021.

Year	No. of blood units donated	No. of blood units issued	No. of platelet units prepared	No. of platelet units issued	No of FFP units prepared	No. of FFP units issued
	Annual data of variables presented as annual total (monthly mean ± SD)					
2019	9507 (792.25 ± 170.82)	9139 (761.58 ± 99.99)	3245 (270.42 ± 140.63)	2562 (213.50 ± 153.81)	2428 (202.33 ± 85.98)	1883 (156.92 ± 43.43)
2020	5117 (426.41 ± 192.43)	5221 (435.08 ± 159.07)	2174 (181.17 ± 48.51)	1030 (85.83 ± 54.25)	1433 (119.42 ± 60.38)	857 (71.42 ± 55.48)
2021	7720 (643.33 ± 323.50)	6353 (529.42 ± 149.22)	4701 (391.75 ± 351.57)	3227 (268.92 ± 414.41)	2369 (197.42 ± 334.88)	1530 (127.50 ± 114.36)
2022	10,184 (848.67 ± 209.29)	8681 (723.42 ± 127.94)	5336 (444.67 ± 201.06)	2982 (248.50 ± 185.46)	3771 (314.25 ± 155.64)	1777 (148.08 ± 47.04)
	Statistical difference between the years 2019, 2020, 2021, and 2022 by Man-Whitney *U* test (*P* value)					
2019 and 2020	.00023	.00005	.15616	.01129	.00508	.00055
2020 and 2021	.01754	.09697	.07861	.29168	.12903	.06301
2019 and 2021	.02319	.00045	.3325	.10725	.01499	.06281
2019 and 2022	.17038	.27218	.01754	.27218	.0499	.17744
2020 and 2022	.00019	.00024	.00036	.00255	.0011	.00161
2021 and 2022	.02319	.00305	.09696	.07861	.00696	.092

FFP = fresh frozen plasma, SD = standard deviation.

### 3.1. Blood supply management

#### 3.1.1. Blood donation

During the study period of the years 2019 to 2022, a total of 32,528 blood donors donated blood, of which 32,209 (99.02%) were male and 319 (0.98%) were female. The male and female donor’s ages were 31.16 ± 8.43 and 33.33 ± 9.50 years, respectively. All the blood donors were non-remunerated and non-commercial. Blood donors include 20,883 (64.2%) voluntary and 11,645 (35.6%) replacement or family donors. The occupational distribution of blood donors includes 13,499 (41.49%) employees of the private sector, 10,376 (31.89%) self-employed, 5205 (16.00%) students, and 3448 (10.6%) government employees.

**Figure 2.**
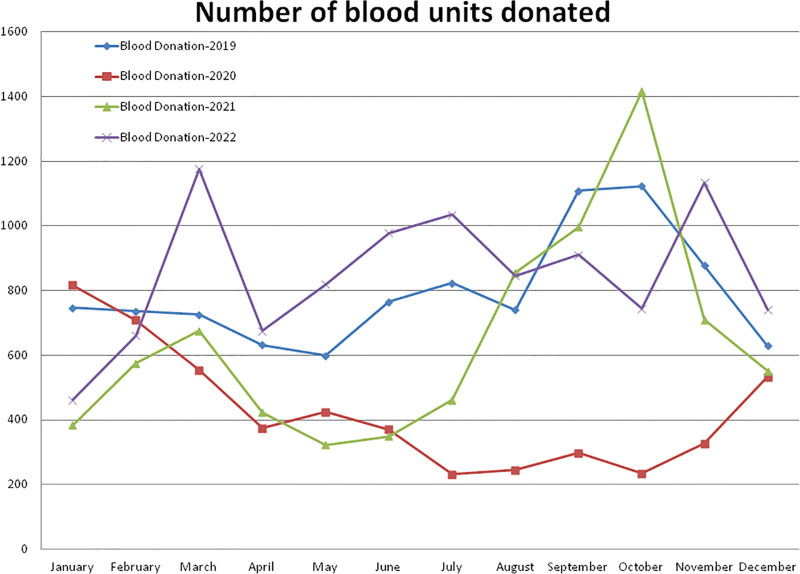
Showing monthly differences due to the COVID-19 pandemic in the monthly trends of the number of blood units donated (collected) during the years 2019, 2020, 2021, and 2022.

Compared to the non-pandemic year 2019, the year 2020 started with a higher (+9.37%, January) number of blood donations, and after the pandemic lockdown it decreased gradually to a maximum difference of –79.16% in the month of October 2020 (Figs. 1 and 2). A total of –46.17% and –18.78% less and +7.12% more blood donation were observed during the years 2020, 2021, and 2022, respectively, compared to the year 2019 (Table 1, Figs. 1 and 2). A total of 5117 blood units were collected in the year 2020, which was significantly lower (*P* = .00023) than the 9507 units during the year 2019. Compared to the year 2019, a higher (+26.18%) number of blood donations was observed in the month of October 2021 and a lower (–54.38%) rate in the month of June 2021. A total of 7720 blood units were collected in the year 2021, which was significantly lower (*P* = .02319) than the year 2019 and +50.20% higher (*P* = .01754) than the year 2020. A total of 10,184 blood units were collected in the year 2022, which was slightly higher than the year 2019 and significantly higher than the years 2020 (*P* = .00019) and 2021 (*P* = .02319). The monthly mean (±SD = standard deviation [SD]) of blood unit collection was 792.25 ± 170.82, 426.41 ± 192.43, 643.33 ± 323.50, and 848.67 ± 209.29 units for the years 2019, 2020, 2021, and 2022, respectively.

### 3.2. Blood component preparation

#### 3.2.1. Platelet (RDP) preparation

**Figure 3.**
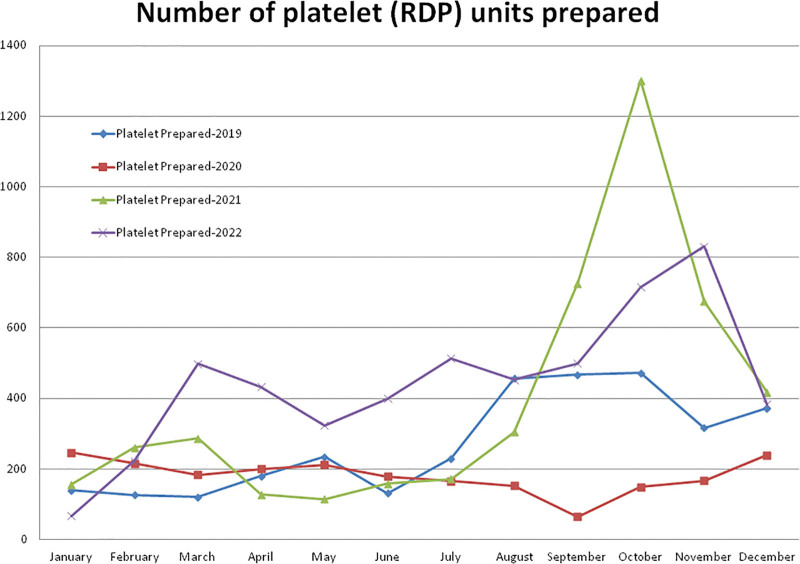
Showing monthly differences due to the COVID-19 pandemic in the monthly trends of the number of platelet units prepared during the years 2019, 2020, 2021, and 2022. RDP = random donor platelet.

Compared to the non-pandemic year 2019, the year 2020 started with a higher (+75.71%, January) number of RDP preparations, and after the pandemic lockdown, it decreased gradually to a maximum difference of –86.08% in the month of September 2020 (Table 1, Figs. 1 and 3). A total of –33.00% less and +44.86% more RDP preparation were observed during the years 2020 and 2021, respectively, compared to the year 2019. A total of 2174 RDP units were prepared in the year 2020, which was lower than the 3245 units during the year 2019. Compared to the year 2019, a higher (+175.64%) number of RDP preparations were observed in the month of October and a lower (–50.85%) rate in the month of May 2021. A total of 4701 units of RDP were prepared in the year 2021, which was +116.23% higher than the year 2020 but not statistically significant higher than the years 2019 and 2020. Compared to the years 2019, 2020, and 2021, a higher number of RDP units (5336) were prepared in the year 2022. The monthly mean (±SD) of RDP preparation was 270.42 ± 140.63, 181.17 ± 48.51, 391.75 ± 351.57, and 444.67 ± 201.06 units for the years 2019, 2020, 2021, and 2022, respectively.

### 3.3. FFP preparation

**Figure 4.**
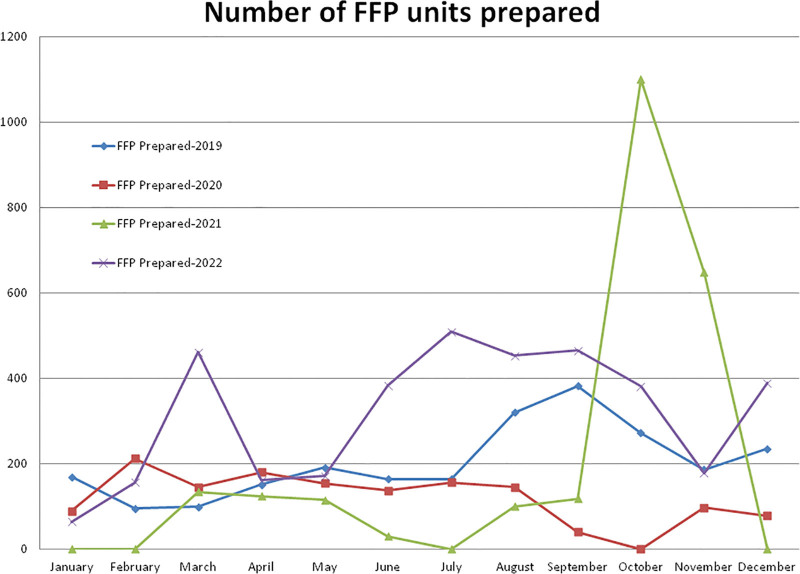
Showing monthly differences due to the COVID-19 pandemic in the monthly trends of FFP units prepared during the years 2019, 2020, 2021, and 2022. FFP = fresh frozen plasma.

Compared to the non-pandemic year 2019, the year 2020 started with a lower (–47.33%, January) number and a +123.15% higher number of FFP preparations in the month of February and after the pandemic lockdown, it decreased gradually to a maximum difference of –100.00% in the month of October 2020 (Figs. 1 and 4). A total of –40.98% and –2.42% less FFP preparation was observed during the years 2020 and 2021, respectively, compared to the year 2019 (Table 1, Figs. 1 and 4). A total of 1433 FFP units were prepared in the year 2020, which was significantly lower (*P* = .0050) than the 2428 units prepared in the year 2019. Compared to the year 2019, a higher (+304.41%) number of FFP preparations were observed in the month of October and a lower (–100.00%) number in the months of January, February, July, and December 2021. A total of 2369 FFP units were prepared in the year 2021, which was significantly (*P* = .0149) lower than the year 2019 and +65.31% higher but not statistically significant (*P* = .1290) than the year 2020. Compared to years 2019, 2020, and 2021, a higher number of FFP units (3771) was prepared in year 2022. The monthly mean (±SD) of FFP preparation was 202.33 ± 85.98, 119.42 ± 60.38, 197.42 ± 334.88, and 314.25 ± 155.64 units for the years 2019, 2020, 2021, and 2022, respectively.

### 3.4. Blood components issued (recipient)

#### 3.4.1. Number of request for blood (whole blood and PRBC) units

**Figure 5.**
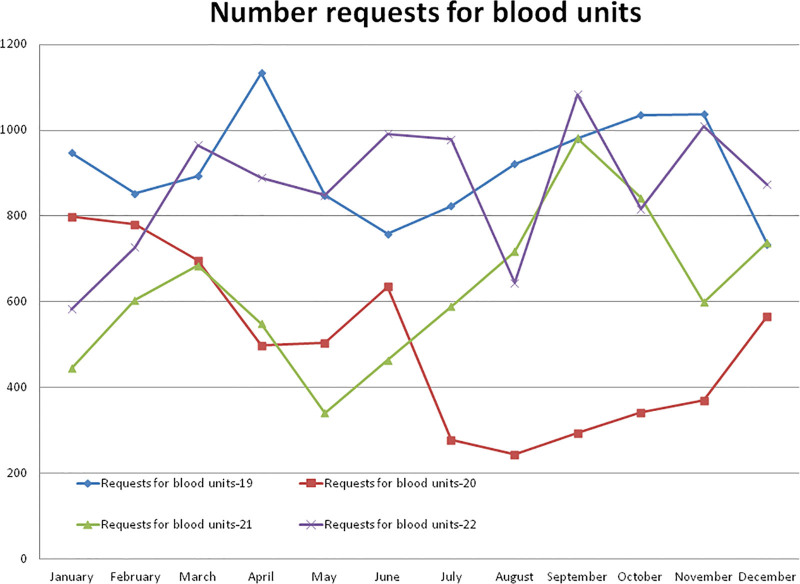
Showing monthly differences due to the COVID-19 pandemic in the monthly trends of the number of requests for blood units during the years 2019, 2020, 2021, and 2022.

Compared to the non-pandemic year 2019, the year 2020 started with a lower (–15.82% in January) number of requests for blood units, and after the pandemic lockdown, it decreased to a maximum difference of –73.53% in the month of August 2020. A total of –45.24% and –31.12% less requests for blood units were observed during the years 2020 and 2021, respectively, compared to the year 2019. A total of 6005 requests for blood units were received in the year 2020, which was significantly lower (*P* = .000) than the 10,968 units during the year 2019 (Table 2 and Fig. 5). Compared to the year 2019, a higher (+0.54%) number of requests for blood units were observed in the month of December and a lower (–59.78%) rate in the month of May 2021. A total of 7554 requests for blood units were received in the year 2021, which was significantly lower (*P* = .000) than the year 2019. A total of 10,413 requests for blood units were received in the year 2022, which was significantly higher than the year 2020 (*P* = .000) and lower but not significant than the year 2019 (*P* = .262). The monthly mean (±SD) of the number of requests for blood units was 914.00 ± 1 19.84, 500.42 ± 197.10, 629.50 ± 177.39, and 867.75 ± 153.45 for the years 2019, 2020, 2021, and 2022, respectively.

**Table 2:** Data of number of request for blood units, number of blood units cross matched, number of request for platelet units, number of platelet units cross matched, number of request for FFP units, number of FFP units cross matched were presented as annual total (monthly mean ± SD) for the year 2019 to 2022.

Year	No. of request for blood units	No. of blood units cross matched	No. of request for platelet units	No. of platelet units cross matched	No. of request for FFP units	No. of FFP units cross matched
	Annual data of variables presented as annual total (monthly mean ± SD)					
2019	10,968 (914.00 ± 119.84)	11,784 (982.00 ± 129.04)	3586 (298.83 ± 215.77)	3658 (304.83 ± 217.23)	2821 (235.08 ± 65.07)	2907 (242.25 ± 65.09)
2020	6005 (500.42 ± 197.10)	6528 (544.00 ± 227.92)	1439 (119.91 ± 75.93)	1539 (128.25 ± 77.58)	1293 (107.75 ± 84.22)	1385 (115.41 ± 87.03)
2021	7554 (629.50 ± 177.39)	7751 (645.91 ± 182.79)	4350 (362.50 ± 559.39)	4413 (367.75 ± 565.33)	2244 (187.0 ± 167.96)	2349 (195.75 ± 175.19)
2022	10,413 (867.75 ± 153.45)	11,320 (943.33 ± 165.53)	4079 (339.91 ± 254.08)	4173 (347.75 ± 258.40)	2663 (221.91 ± 70.56)	2797 (233.08 ± 72.69)
	Statistical difference between the years 2019, 2020, 2021, and 2022 by Man-Whitney *U* test (*P* value)					
2019 and 2020	<.001	<.001	.011	.014	<.001	<.001
2020 and 2021	.079	.170	.343	.333	.079	.079
2019 and 2021	<.001	<.001	.097	.097	.044	.056
2019 and 2022	.263	.364	.272	.272	.177	.226
2020 and 2022	<.001	<.001	.003	.004	.002	.001
2021 and 2022	.003	<.001	.079	.079	.079	.079

SD = standard deviation.

#### 3.4.2. Number of blood units (whole blood and PRBC) cross-matched

**Figure 6.**
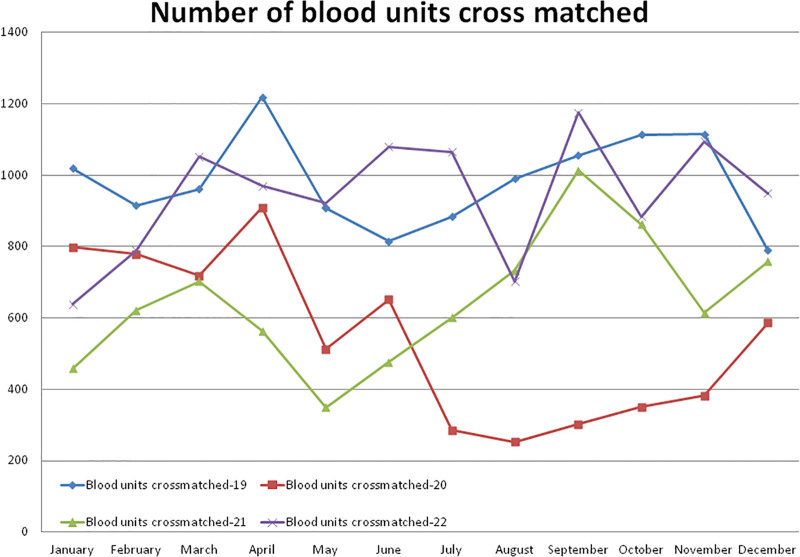
Showing monthly differences due to the COVID-19 pandemic in the monthly trends of the number of cross-sectional blood units during the years 2019, 2020, 2021, and 2022.

Compared to the non-pandemic year 2019, the year 2020 started with a lower (–21.68% in January) number of blood units cross-matched, and after the pandemic lockdown, it decreased to a maximum difference of –74.44% in the month of August 2020. A total of –44.6% and –34.22% less cross-matching blood units were observed in 2020 and 2021, respectively, compared to 2019. A total of 6528 cross-matched blood units were observed in the year 2020, which was significantly lower (*P* = .000) than the 11,784 units observed in the year 2019 (Table 2 and Fig. 6). Compared to the year 2019, the lowest (–61.56%) number of blood units cross-matched was observed in the month of May 2021. A total of 7751 blood units were cross-matched in the year 2021, which was significantly lower (*P* = .000) than the year 2019. A total of 11,320 blood units were cross-matched in the year 2022, which was significantly higher than the year 2020 (*P* = .000) and lower but not significantly lower than the year 2019 (*P* = .364). The monthly mean (±SD) of the number of blood units cross-matched was 982.00 ± 129.04, 544.00 ± 227.92, 645.91 ± 182.79, and 943.33 ± 165.53 for the years 2019, 2020, 2021, and 2022, respectively.

### 3.5. Blood (whole blood and PRBC) units issued to recipients

**Figure 7.**
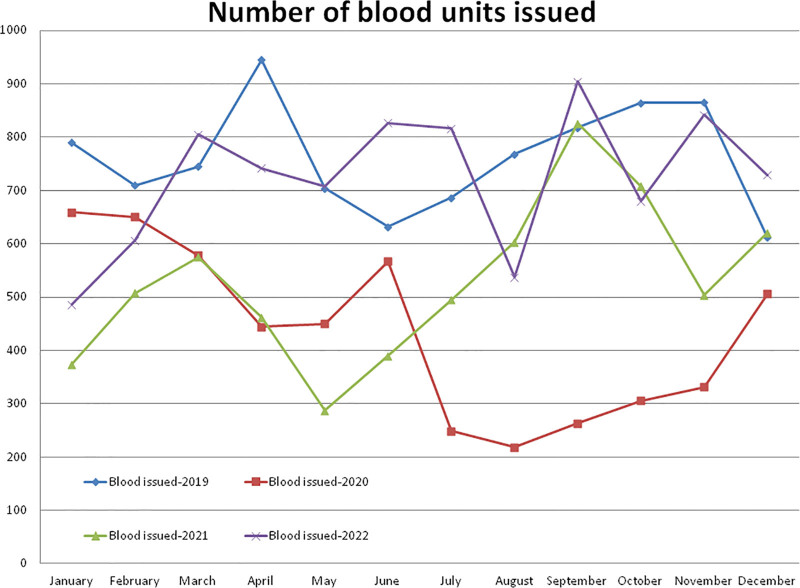
Showing monthly differences due to COVID-19 pandemic in the monthly trends of number of blood units issued during the years 2019, 2020, 2021, and 2022.

Compared to the non-pandemic year 2019, the year 2020 started with a lower (–8.45%, January) number of blood recipients and after the pandemic lockdown, it decreased to a maximum difference of –71.61% in the month of August 2020 (Figs. 1 and 7). A total of –42.87% and –30.48% fewer blood unit recipients were observed during the years 2020 and 2021, respectively, compared to the year 2019 (Table 1, Figs. 1 and 7). A total of 5221 blood units were issued in the year 2020, which was significantly lower (*P* = .0000) than the 9139 units during the year 2019. Compared to the year 2019, a higher (+1.47%) number of blood unit recipients were observed in the month of December and a lower (–59.23%) rate in the month of May 2021. A total of 6353 blood units were issued in the year 2021, which was significantly lower (*P* = .0004) than the year 2019 and was +21.68% higher but not statistically significant (*P* = .0969) than the year 2020. A total of 8681 blood units were issued in the year 2022, which was significantly higher than the years 2020 (*P* = .00024) and 2021 (*P* = .00305) and lower but not significantly lower than the year 2019 (*P* = .27218). The monthly mean (±SD) of blood units issued to the recipients was 761.58 ± 99.99, 435.08 ± 159.07, 529.42 ± 149.22, and 723.42 ± 127.94 units for the years 2019, 2020, 2021, and 2022, respectively.

#### 3.5.1. Number of requests for platelet (RDP) units

**Figure 8.**
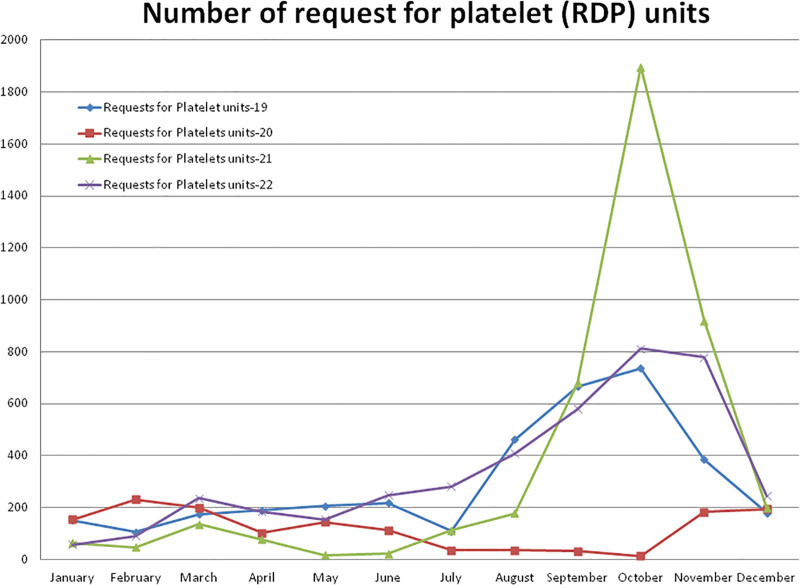
Showing monthly differences due to COVID-19 pandemic in the monthly trends of number of request for RDP units during the year 2019, 2020, 2021, and 2022. RDP = random donor platelet.

Compared to non-pandemic year 2019, in year 2020, the higher (+117.92%) number of requests for RDP units were observed in the month of February and after the pandemic lockdown, it decreased to a maximum difference of –98.10% in the month of October 2020 (Figs. 1 and 6). A total of –59.87% less and +21.31% and +13.74% more requests for RDP units were observed during the years 2020, 2021, and 2022, respectively, compared to the year 2019. A total of 1439 requests for RDP units were received in the year 2020, which was significantly (*P* = .011) lower than the 3586 units during the year 2019 (Table 2 and Fig. 8). Compared to the year 2019, a higher (+157.6%) number of requests for RDP units were observed in the month of October and a lower (–91.3%) number in the month of May 2021. A total of 4350 requests for RDP units were received in the year 2021, which was higher but not statistically significant than the years 2020 (*P* = .343) and 2019 (*P* = .096). A total of 4079 requests for RDP units were received in 2022, which was higher but not statistically significant than the year 2019 (*P* = .272). The monthly mean (±SD) of requests for RDP units were 298.83 ± 215.77, 119.91 ± 75.93, 362.50 ± 559.39, and 339.91 ± 254.08 for the years 2019, 2020, 2021, and 2022, respectively.

#### 3.5.2. Number of platelet (RDP) units cross matched

**Figure 9.**
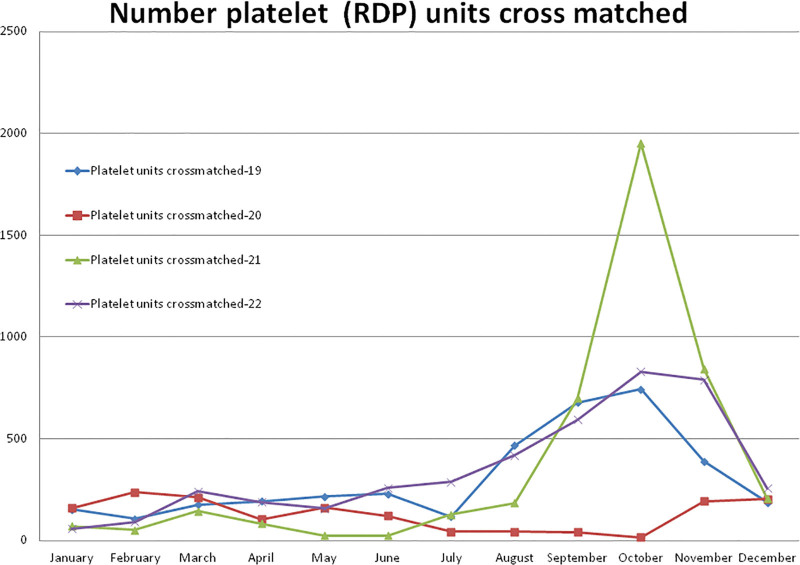
Showing monthly differences due to COVID-19 pandemic in the monthly trends of number of cross match of RDP units during the year 2019, 2020, 2021 and 2022. RDP = random donor platelet.

Compared to non-pandemic year 2019, in year 2020, a higher (+122.43%) number of cross-match RDP units were observed in the month of February and after the pandemic lockdown, it decreased to a maximum difference of –97.84% in the month of October 2020 (Figs. 1 and 6). A total of –57.92% less and +20.63% and +14.07% more cross-match of RDP units were observed during the years 2020, 2021, and 2022, respectively, compared to the year 2019. A total of 1539 cross-matches of RDP units were observed in the year 2020, which was significantly (*P* = .014) lower than the 3658 units during the year 2019 (Table 2 and Fig. 9). Compared to the year 2019, a higher (+162.23%) number of cross-match RDP units were observed in the month of October and a lower (–89.52%) number in the month of May 2021. A total of 4413 cross-matches of RDP units were observed in the year 2021, which was higher but not statistically significant than the years 2020 (*P* = .342) and 2019 (*P* = .096). A total of 4173 cross-matches of RDP units were observed in 2022, which was higher but not statistically significant than the year 2019 (*P* = .272). The monthly mean (±SD) of cross-matching RDP units were 304.83 ± 217.23, 128.25 ± 77.58, 367.75 ± 565.33, and 347.75 ± 258.40 for the years 2019, 2020, 2021, and 2022, respectively.

#### 3.5.3. Platelet (RDP) units issued to recipients

**Figure 10.**
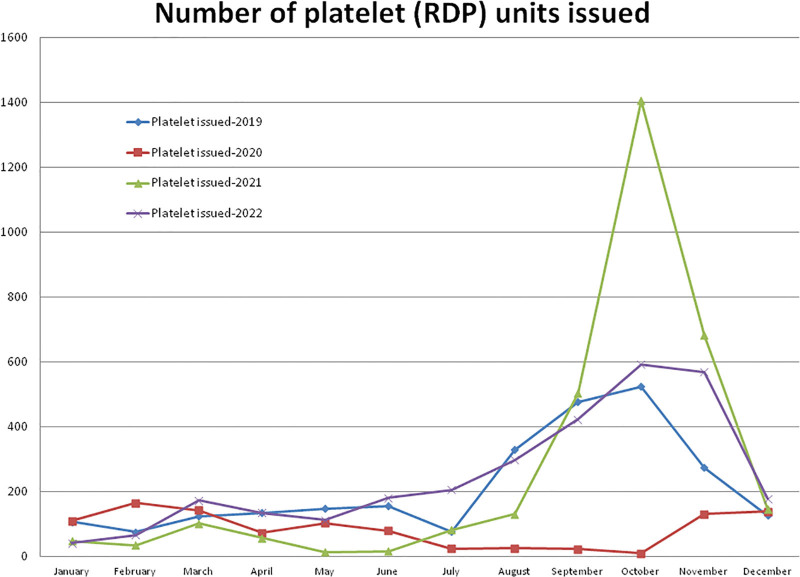
Showing monthly differences due to COVID-19 pandemic in the monthly trends of number of RDP units issued during the years 2019, 2020, 2021, and 2022. RDP = random donor platelet.

Compared to the non-pandemic year 2019, in 2020, the higher (+117.11%) number of RDP recipients was observed in the month of February and after the pandemic lockdown, it decreased to a maximum difference of –98.09% in the month of October 2020 (Figs. 1 and 10). A total of –59.79% fewer and +25.95% more RDP unit recipients were observed during the years 2020 and 2021, respectively, compared to the year 2019 (Table 1, Figs. 1 and 10). A total of 1030 RDP units were issued in the year 2020, which was significantly (*P* = .0112) lower than the 2562 units during the year 2019. Compared to the year 2019, a higher (+168.13%) number of RDP unit recipients were observed in the month of October and a lower (–90.54%) number in the month of May 2021. A total of 3227 RDP units were issued in the year 2021, which was +213.30% higher than the year 2020 but not statistically significant compared to the years 2020 (*P* = .2916) and 2019 (*P* = .1072). A total of 2982 RDP units were issued in the year 2022, which is significantly higher than the year 2020 and not significantly higher than the year 2019 (*P* = .27218) and lower than the year 2021 (*P* = .07861). The monthly mean (±SD) of RDP units issued to recipients was 213.50 ± 153.81, 85.83 ± 54.25, 268.92 ± 414.41, and 248.50 ± 185.46 units for the years 2019, 2020, 2021, and 2022, respectively.

#### 3.5.4. Number of requests for FFP units

**Figure 11.**
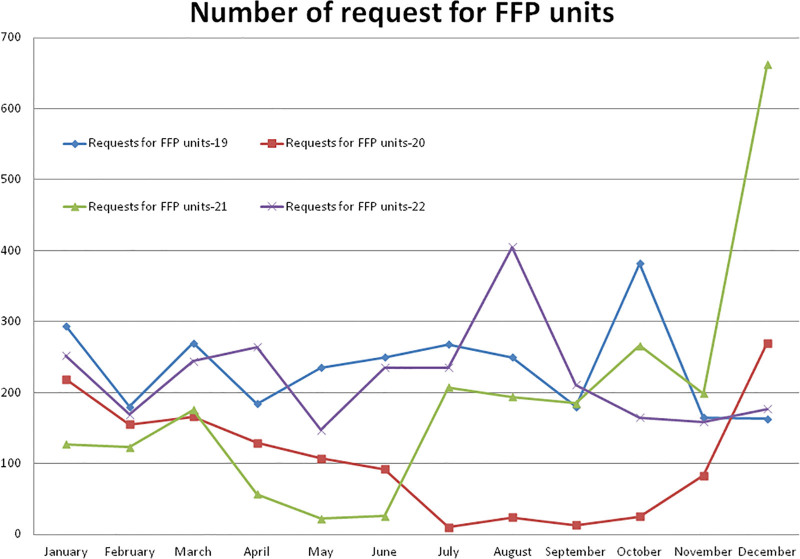
Showing monthly differences due to COVID-19 pandemic in the monthly trends of request of FFP units during the years 2019, 2020, 2021, and 2022. FFP = fresh frozen plasma.

Compared to the non-pandemic year 2019, the year 2020 started with a lower (–25.51%, January) number of requests for FFP units and after the pandemic lockdown, it decreased to a maximum difference of –96.26% in the month of July and then increased to +65.64% in the month of December 2020. A total of –54.16%, –20.45%, and –5.60% less requests for FFP units were observed during the years 2020, 2021, and 2022, respectively, compared to the year 2019. A total of 1293 requests for FFP units were observed in the year 2020, which was significantly (*P* = .000) lower than the 2821 units during the year 2019 (Table 2 and Fig. 11). Compared to the year 2019, a higher (+306.13%) number of requests for FFP units were received in the month of December and a lower (–90.64%) in the month of May 2021. A total of 2244 requests for FFP units were observed in 2021, which was significantly (*P* = .044) lower than the year 2019 and higher but not statistically significant (*P* = .078) than the year 2020. A total of 2663 requests for FFP units were observed in 2022, which was significantly higher (*P* = .001) than the year 2020. The monthly mean (±SD) of requests for FFP units was 235.08 ± 65.07, 107.75 ± 84.22, 187.0 ± 167.96, and 221.91 ± 70.56 units for the years 2019, 2020, and 2021, respectively.

#### 3.5.5. Number of FFP units cross matched

**Figure 12.**
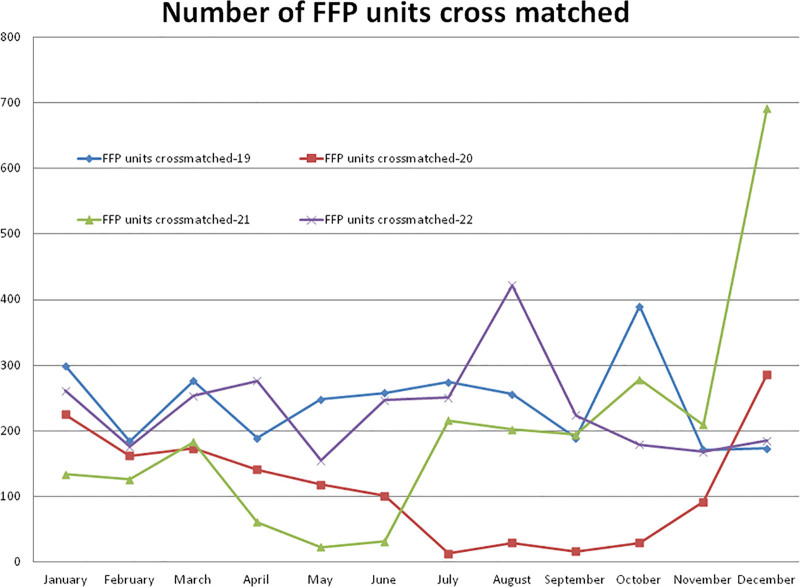
Showing monthly differences due to COVID-19 pandemic in the monthly trends of cross match of FFP units during the years 2019, 2020, 2021, and 2022. FFP = fresh frozen plasma.

Compared to the non-pandemic year 2019, the year 2020 started with a lower (–24.74%, January) number of cross-match FFP units, and after the pandemic lockdown, it decreased to a maximum difference of –95.25% in the month of July and then increased to +65.31% in the month of December 2020. A total of –52.34%, –19.19%, and -3.78% less cross-match of FFP units were observed during the years 2020, 2021, and 2022, respectively, compared to the year 2019. A total of 1385 FFP units were observed in 2020, which was significantly (*P* = .000) lower than the 2907 units observed in 2019 (Table 2 and Fig. 12). Compared to the year 2019, a higher (+300.00%) number of cross-match FFP units were observed in the month of December and a lower (–90.72%) in the month of May 2021. A total of 2349 cross-matches of FFP units were observed in the year 2021, which was not statistically significant but lower than the year 2019 (*P* = .056) and higher (*P* = .078) than the year 2020. A total of 2797 FFP unit cross-matches were observed in 2022, which was significantly higher (*P* = .001) than the year 2020. The monthly mean (±SD) of cross-matching FFP units was 242.25 ± 65.09, 115.41 ± 87.03, 195.75 ± 175.19, and 233.08 ± 72.69 for the years 2019, 2020, and 2021, respectively.

### 3.6. FFP units issued to recipients

**Figure 13.**
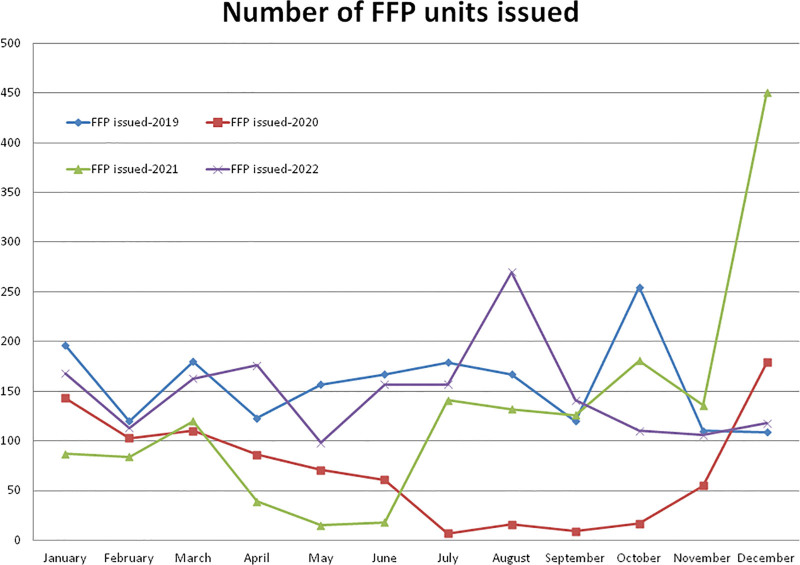
Showing monthly differences due to COVID-19 pandemic in the monthly trends of FFP units issued during the years 2019, 2020, 2021, and 2022. FFP = fresh frozen plasma.

Compared to the non-pandemic year 2019, the year 2020 started with a lower (–27.04%, January) number of FFP recipients, and after the pandemic lockdown, it decreased to a maximum difference of –96.09% in the month of July and then increased to +64.22% in the month of December 2020 (Figs. 1 and 13). A total of –54.48% and –18.75% less number of FFP unit recipients were observed during the years 2020 and 2021, respectively, compared to the year 2019 (Table 1, Figs. 1 and 13). A total of 857 FFP units were issued in the year 2020, which was significantly (*P* = .0005) lower than the 1883 units during the year 2019. Compared to the year 2019, a higher (+313.76%) number of FFP unit recipients were observed in the month of December and a lower (–90.45%) in the month of May 2021. A total of 1530 FFP units were issued in 2021, which was lower but not statistically significant (*P* = .0628) than the year 2019 and higher (+78.52%) but not statistically significant (*P* = .0630) than the year 2020. A total of 1777 FFP units were issued in 2022, which was significantly higher (*P* = .00161) than the year 2020. The monthly mean (±SD) of FFP units issued to recipients was 156.92 ± 43.43, 71.42 ± 55.48, 127.50 ± 114.36, and 148.08 ± 47.04 units for the years 2019, 2020, and 2021, respectively.

Additional COVID-19 cope-up measures adopted as precautionary measures at our blood center include:

1. Staff and blood donor safety:
  - To facilitate staggered in-flow, donors were encouraged to make appointments for blood donation.
  - It was mandatory for all persons to wear face masks, undergo temperature screening, use hand sanitizer and maintain social distance to access the blood center.
  - The blood donor consent form was modified for additional COVID-19 contact and travel history of donor.
  - Social distancing of 2 m maintained between donor couches which was covered with disposable sheets and replaced after every donation.
  - The blood bank was kept functioning with 50% strength of the staff in 2 shifts per day on rotation of every 3 days.
  - Staff having COVID-19 contact, get quarantined and having COVID-19 positive test results get isolated as per given guideline.
2. Blood donor recruitment:
  - The information, education, and communication (IEC) materials were circulated to blood donation camp organizers and blood donors. It includes blood donor eligibility (inclusion) and deferral (exclusion) criteria, COVID-19 signs and symptoms, COVID-19 protocol for blood donation and benefits of blood donation, addressing the constant blood need for patients on regular transfusion support.
  - Blood donor inclusion criteria included in addition: no travel history of blood donor in COVID-19 infected area within the past 28 days, no contact history with suspected or confirmed COVID-19–infected person within the past 28 days and who were neither suspected nor confirmed within the past 28 days to have COVID-19 infection.
  - Blood donor exclusion criteria included in addition: exclusion or deferral period of blood donor includes a period of 28 days following recovery, contact with a COVID-19–infected person, and travel history of COVID-19–infected area and respiratory symptoms.
  - Recruitment and motivation of health care personal, medical and paramedical students of our hospital for blood donation.
  - The reminder calls request to registered regular voluntary blood donors after the completion of their post donation interval period.
  - During the pandemic lockdown, a blood donor road pass was given to the donor to travel to and from the blood bank to donate blood.
3. Blood supply management
  - The temporary suspension of all the non-emergency surgical cases in the hospital during the first, second, and third waves of the pandemic. Clinicians were requested to follow restrictive transfusion practices, utilize possible blood alternatives, such as oral or parenteral iron and erythropoiesis-stimulating agents if applicable and transfuse blood based on the assessment of risk versus benefit for the patient.
  - Daily monitoring of the blood stock (inventory) and issue of blood components to avoid the unnecessary prolonged reservation of blood components for a particular patient.

## 4. DISCUSSION

During the COVID-19 pandemic, recruitment of safe and healthy blood donors remained the primary challenge for blood banks and transfusion services worldwide. Despite the challenges posed by the COVID-19 pandemic, blood banks continued their services to strive to provide an uninterrupted supply of blood to meet the ongoing need of blood for patients. Moreover, the guidelines and recommendations of WHO,^14^ NACO, NBTC,^13^ AABB,^15^ and CDC^16^ paved the way during the hard times of the COVID-19 pandemic to maintain a safe blood transfusion chain.

The blood supply and inventory management at our blood bank revealed that the COVID-19 pandemic drastically affected the blood transfusion services during the pandemic lockdown. Compared to the non-pandemic year 2019, the year 2020 showed a minimal rise in blood collection, component preparation, and demand for platelets with a lower demand for blood (WB and PCV) and FFP during the pre-lockdown phase. After the pandemic lockdown in March 2020, all the variables significantly decreased gradually (Tables 1 and 2 and Figs. 1–13). During the pandemic lockdown in 2020, the annual decrease in blood collection, request for blood components, cross-match, and issue was –46.17%, –45.24%, –44.6%, and –42.87%, respectively, as compared to 2019. The annual decrease in RDP preparation, request for RDP, cross-match, and issue was –33.00%, –59.87%, –57.92%, and –59.79%, respectively. The annual decrease in FFP preparation, request for FFP, cross-match, and issue was –40.98%, –54.16%, –52.34%, and –54.48%, respectively. Similar to our results, a reduction in blood collection, demand and issue of –33%, –31.8%, and –32.3%, respectively, was observed by Kandasamy et al^4^ in southern Karnataka, India. A reduced blood donation of –67% was observed by Wang et al in the Zhejiang Province of China, while –30% drop in blood donation was observed by the Canadian Blood Services during the pandemic period.^8–21^

Compared to the year 2020, in the year 2021, due to relaxations in lockdown, more patients were admitted for all types of cases, including surgery, and there was an annual increase in blood collection and issues (+50.20% and +21.68%, respectively). An annual increase in FFP preparation and issue was seen (+65.31% and +78.52%, respectively). There was an increase in the demand for platelets (RDP and SDP) due to the seasonal dengue outbreak. All variables of blood transfusion services were increased gradually; the annual increase in RDP preparation and issue was +116.23% and +213.30%, respectively. Compared to pandemic year 2020, in 2022 a significantly higher number of blood collections, blood component preparation, requests for blood components, cross-matches, and issues were observed (Tables 1 and 2 and Figs. 1–13). No significant difference was observed in blood collection, blood component preparation, requests for blood components, cross-matching, and issuing between the non-pandemic years of 2019 and 2022. Similar to our results, the studies on the previous SARS epidemic in 2003 and the present COVID-19 pandemic showed that it caused an imbalance between the blood demand and supply due to a significant drop in blood donation.^4,18–25^

The demographic characteristics of blood donors at our center show a higher proportion of males (99.02%) and a lower (0.98%) proportion of females. All the blood donors were non-remunerated and non-commercial. The proportions of voluntary and replacement (or family) donors were 64.2% and 35.8%, respectively. The occupational distribution of blood donors includes 41.49% employees of the private sector, 31.89% self-employed individuals, 16.00% students, and 10.6% government employees. We observed that the reduction in blood donation and blood utilization at our center was caused by factors related to the COVID-19 pandemic, such as lockdown, restrictions on public movement, quarantine period, social distancing, and restrictions on holding outdoor blood donation camps to contain the spread of COVID-19 infections and suspend elective surgeries. Similar to our observation, these factors had affected blood transfusion services globally.^13,26,27^ The reduced blood donation led us to recruit and motivate health care personnel, medical, and paramedical students of our hospital, and register regular voluntary blood donors to donate blood. During the pandemic lockdown, a blood donor road pass was given to the donor to travel to and from the blood bank to donate blood. It increased in-house blood donation camps, repeat and first time donors during the pandemic. Similar finding has been reported by Kandasamy et al^4^ and Wang et al.^18^ To cater the need of blood during pandemic crisis donor education and awareness had played important role, we circulated IEC to blood donation camp organizers and potential blood donors, through various platform and social media, which played an important role in increasing the blood donation during the pandemic crisis. Similar communication strategies played a good role across the globe.^4,23–29^ Various COVID-19 cope-up measures which were adopted at our center, helped in balancing the blood demand and supply during the pandemic crisis. Similar strategies adopted in other countries which proved to be affective.^4,23–29^

## 5. CONCLUSION

Our study results show that the COVID-19 pandemic has significantly affected blood transfusion services at our blood bank. The adopted coping strategies to maintain the safe and uninterrupted blood transfusion chain at our blood bank gave us lessons for future preparedness if faced with a similar situation.
